# Correction: Neural signatures of vigilance decrements predict behavioural errors before they occur

**DOI:** 10.7554/eLife.91529

**Published:** 2023-08-08

**Authors:** Hamid Karimi-Rouzbahani, Alexandra Woolgar, Anina N Rich

**Keywords:** Human

Karimi-Rouzbahani H, Woolgar A, Rich AN. 2021. Neural signatures of vigilance decrements predict behavioural errors before they occur. *eLife* **10**:e60563. doi: 10.7554/eLife.60563.

Published 8 April 2021

In the decoding *distance to object* analyses on correct trials, classifiers were trained and tested using data from across timepoints (since each distance was a bin spanning multiple timepoints) and trials. Signal samples from all trials and timepoints within each distance were randomly allocated to one of the 10 folds to be used in the 10-fold cross-validation process. This meant that, on any given iteration of cross-validation, a single sample was never in both the training and the testing set, but samples in the training and testing set could be drawn from different timepoints of the same experimental trial. Autocorrelation in the data over time could therefore have inflated the decoding accuracy. To correct this, we have re-run the analysis decoding *distance to object* ensuring that training and testing sets drew from independent trials, and re-ran all analyses that drew on these decoding results. Analyses that did not decode *distance to object* are not affected, and analyses decoding *distance to object* on error trials are also not affected.

We replicate our main finding of being able to use neural patterns to predict the occurrence of errors, along with all the effects of attention. The interaction indicating decreased distance decoding for late vs early blocks in monitoring but not active conditions has decreased in strength from being evident (BF >3) at two consecutive time-windows to one (BF = 6.7) and there is no longer a main effect of target frequency. The link we reported between behaviour and informational connectivity is no longer present.

This necessitated the changes outlined below. Underline indicates differences between corrected and original text.

[**Abstract**]

[*Corrected text: #1*]

There was subtle evidence of this also in the neural decoding using Magnetoencephalography: for one time-window (of 80ms) coding of critical information declined more during monitoring versus active conditions.

[*Original text: #1*]

This was mirrored in neural decoding using Magnetoencephalography: coding of critical information declined more during monitoring versus active conditions along the experiment.

[**Results: Effects of Target Frequency on critical** ***distance to object*** **information**]

[*Corrected text:* #1]

The same analysis for the representation of the task-relevant *distance to object* information showed strong evidence for a main effect of Attention (BF >10; Bayes factor ANOVA) at all 15 distances, no effect of Time on Task (BF <0.3; Bayes factor ANOVA) at any of the distances, and an interaction between Time on Task and Target Frequency at one of the distances (BF = 6.7, Figure 3B).

[*Original text:* #1]

The same analysis for the representation of the task-relevant *distance to object* information showed strong evidence for a main effect of Attention (BF >10; Bayes factor ANOVA) at all 15 distances, moderate or strong evidence for a main effect of Time on Task (BF >3; Bayes factor ANOVA) at eight of the earlier distances, and an interaction between Time on Task and Target Frequency at two of these distances (Figure 3B).

[*Corrected text:* #2]

[--deleted text--]

[*Original text:* #2]

The main effect of Time on Task reflected decreased decoding in later blocks (compare dashed lines to solid lines in Figure 3B).

[*Corrected text:* #3]

The interaction between Target Frequency and Time on Task at distance 13 (time-window: 160–240ms after stimulus onset, BF = 6.7) reflected opposite effects of time on task in the Active and Monitoring conditions. In Active blocks, there was moderate evidence that coding was stronger in late blocks than in early blocks (BF = 3.1), whereas in the Monitoring condition, decoding declined with time and was weaker in late than in easy blocks (BF = 4.3). However, as there was only moderate evidence for this interaction at one of the time-windows, we do not overinterpret it. Decoding of attended information tended to be lower in late compared to early Monitoring blocks (Figure 3B lower panel red dotted line) in several time-windows across the trial, which may echo the behavioural pattern of performance (Figure 2). As there was moderate evidence for no interaction between Attention and Target Frequency (BF <0.3, 2-way Bayes factor ANOVA) except for distance 6 (BF = 3.3; no consistent pattern (insufficient evidence for pairwise comparisons: BFs 2.4–2.8)), no interaction between Attention and Time on Task (BF <0.3, 2-way Bayes factor ANOVA) or simultaneously between the three factors (BF <0.3, 3-way Bayes factor ANOVA), we do not show those statistical results in the figure.

[*Original text:* #3]

Finally, the interaction between Target Frequency and Time on Task can be seen when comparing the solid to the dashed lines in blue and red colours, separately, and suggests a bigger decline in decoding in Monitoring compared to Active conditions. Note that as there was moderate evidence for no interaction between Attention and Target Frequency or between Attention and Time on Task (BF <0.3, 2-way Bayes factor ANOVA) or simultaneously between the three factors (BF <0.3, 3-way Bayes factor ANOVA), we do not show those statistical results in the figure.

[*Corrected text:* #4]

Specifically, for our crucial *distance to object* data, the main effect of Attention remained after eye-artefact removal, replicating our initial pattern of results. Moderate evidence (BF = 4.2) for an interaction between Target Frequency and Time on Task was also found, but now at distance 6 instead of distance 13. This interaction again reflected a larger effect of Time on Task in Monitoring compared to Active blocks (Monitoring: weaker coding in late relative to early blocks (BF = 3.1); Active: insufficient evidence for change in coding from early to late (BF = 2.0)).

[*Original text:* #4]

Specifically, for our crucial *distance to object* data, the main effects of Attention and Time on Task and the key interaction between Target Frequency and Time on Task remain after eye-artefact removal, replicating our initial pattern of results.

[*Corrected text:* #5]

Thus, although the results are similar with and without standard eye-artefact removal, it is impossible to fully rule out all potential eye movement effects.

[*Original text:* #5]

Thus, although the results replicate with and without standard eye-artefact removal and we find no evidence for eye-related measures to explain our main vigilance effects, it is impossible to fully rule out all potential eye movement effects.

[*Corrected text:* #6]

Together, these results suggest that while vigilance conditions had little or no impact on coding of the *direction of approach*, they did impact the critically task-relevant information about the *distance* of the dot from the object, albeit only for one 80ms time-window. In this time-window, coding declined as time on task increased specifically when the target events happened infrequently, forming a possible neural correlate for our behavioural vigilance decrements.

[*Original text:* #6]

Together, these results suggest that while vigilance conditions had little or no impact on coding of the *direction of approach*, they did impact the critically task-relevant information about the *distance* of the dot from the object. Coding of this information declined as the time on the task increased and this effect was more pronounced when the target events happened infrequently, forming a neural correlate for our behavioural vigilance decrements.

[*Corrected figure: Figure 3B*]

**Figure 1:**
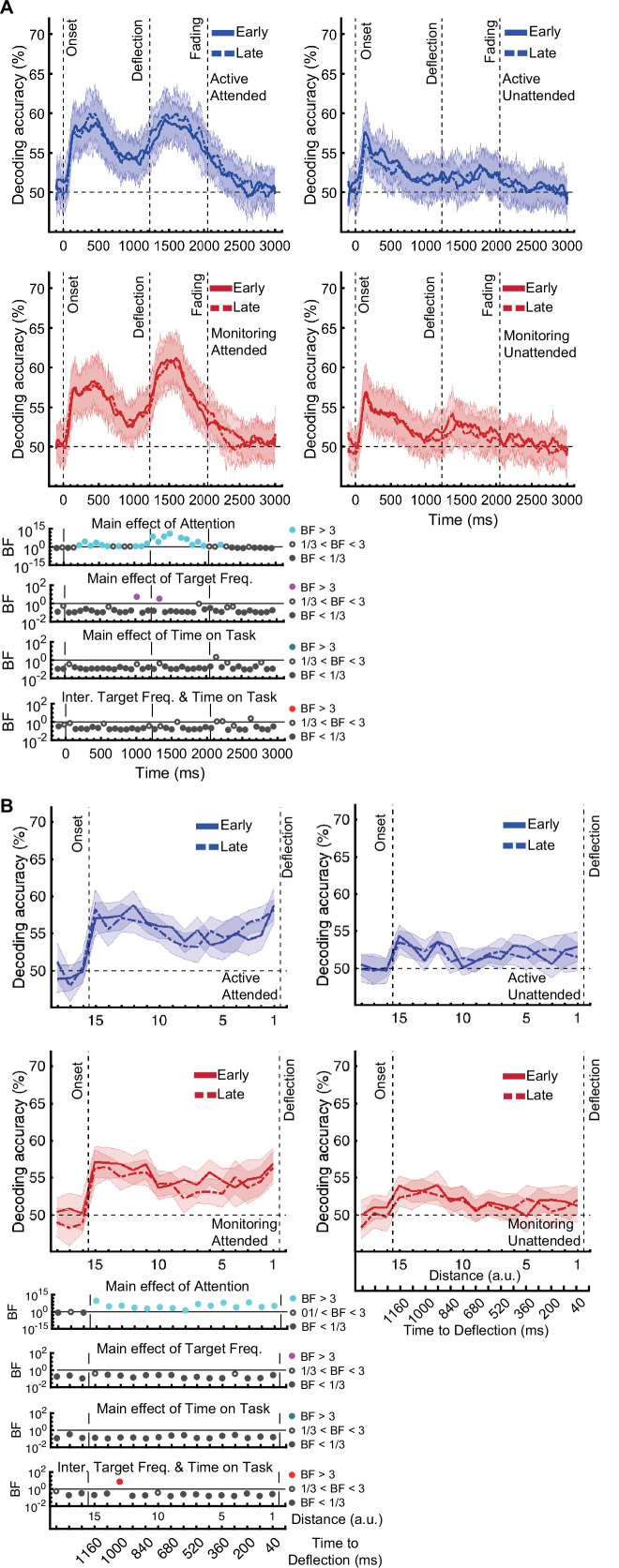


[*Original figure: Figure 3*]

**Figure 2:**
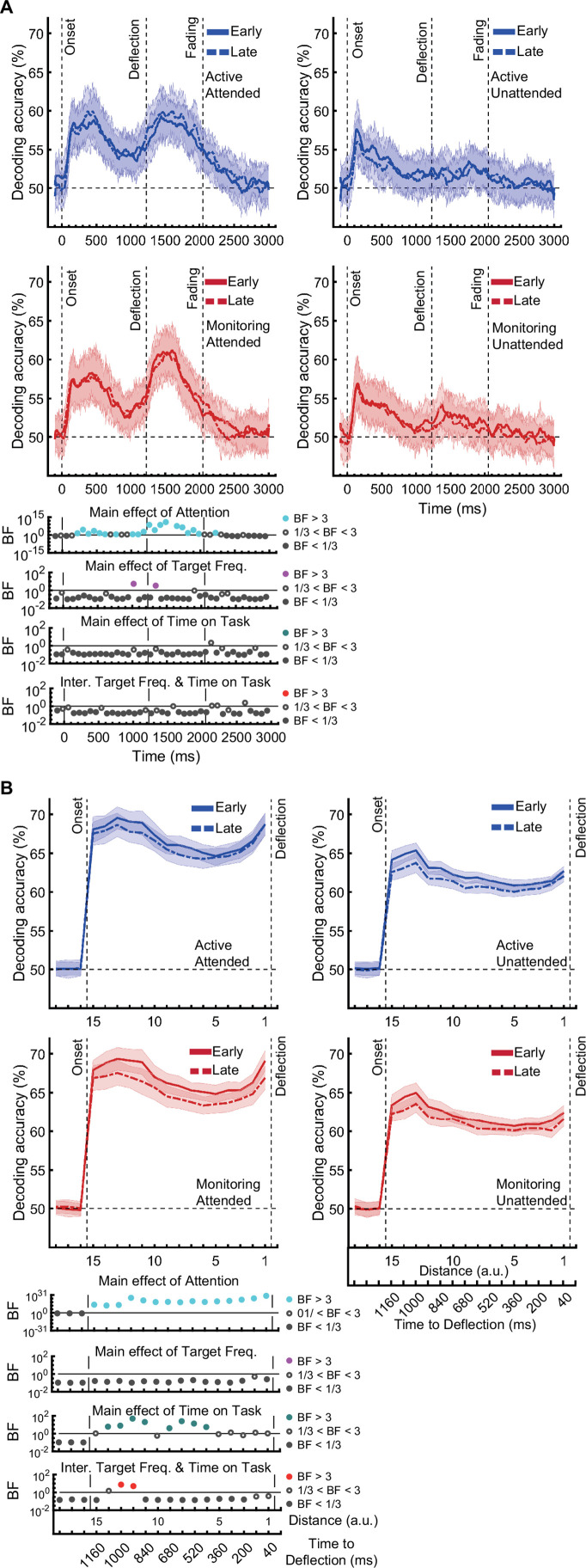


[*Corrected figure: Figure 3—figure supplement 1B*]

**Figure 3:**
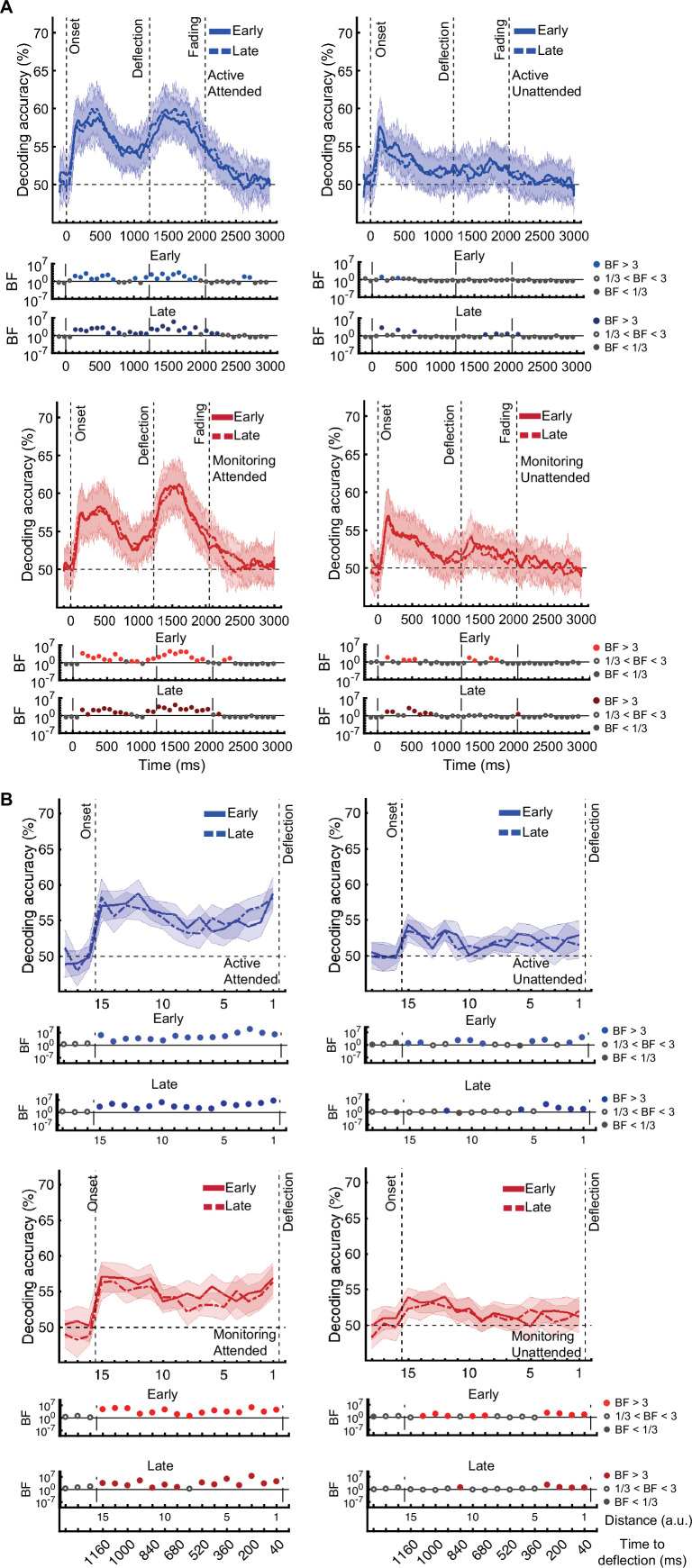


[*Original figure: Figure 3—figure supplement 1*]

**Figure 4:**
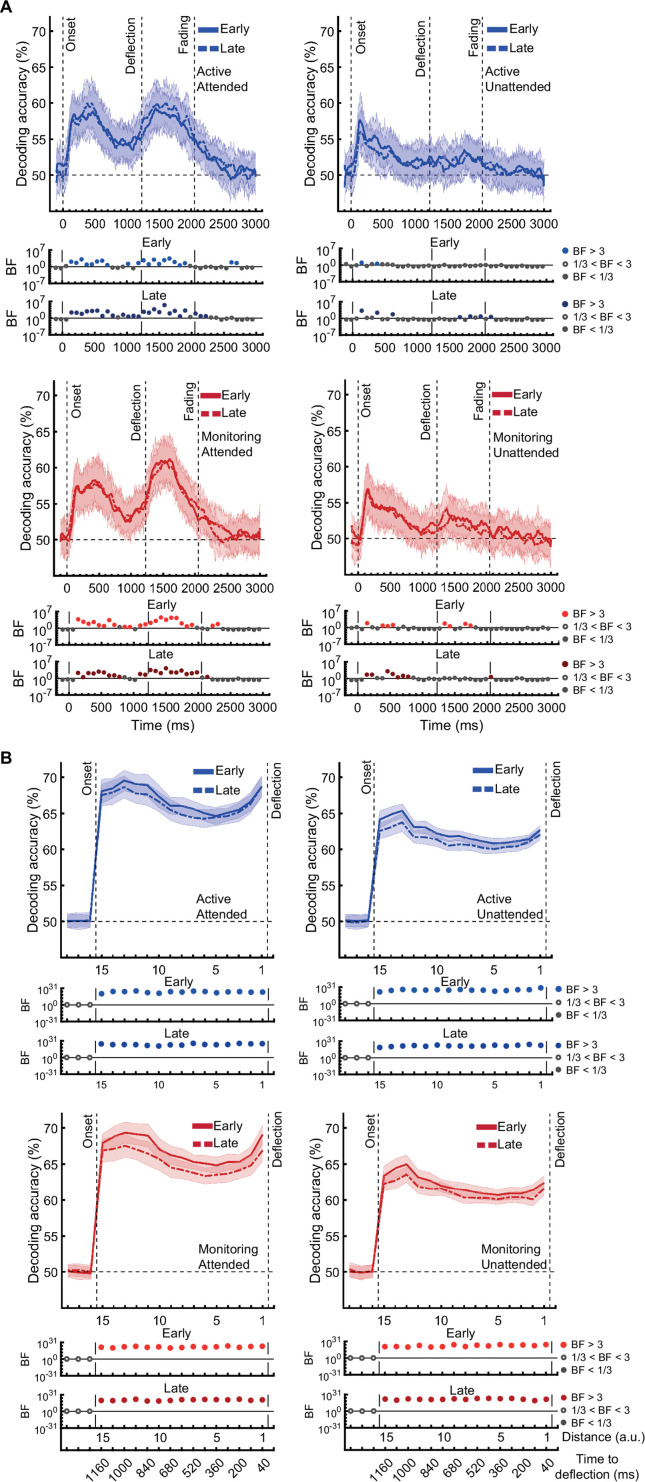


[*Corrected figure: Figure 3—figure supplement 2B*]

**Figure 5:**
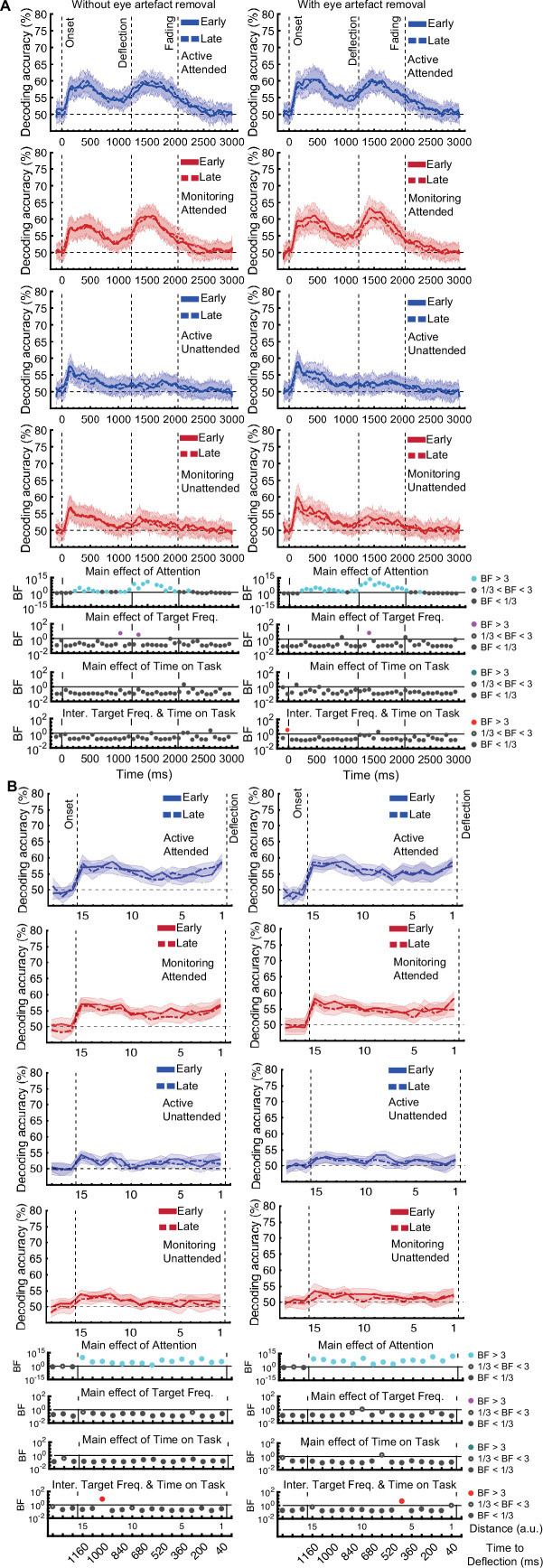


[*Original figure: Figure 3—figure supplement 2*]

**Figure 6:**
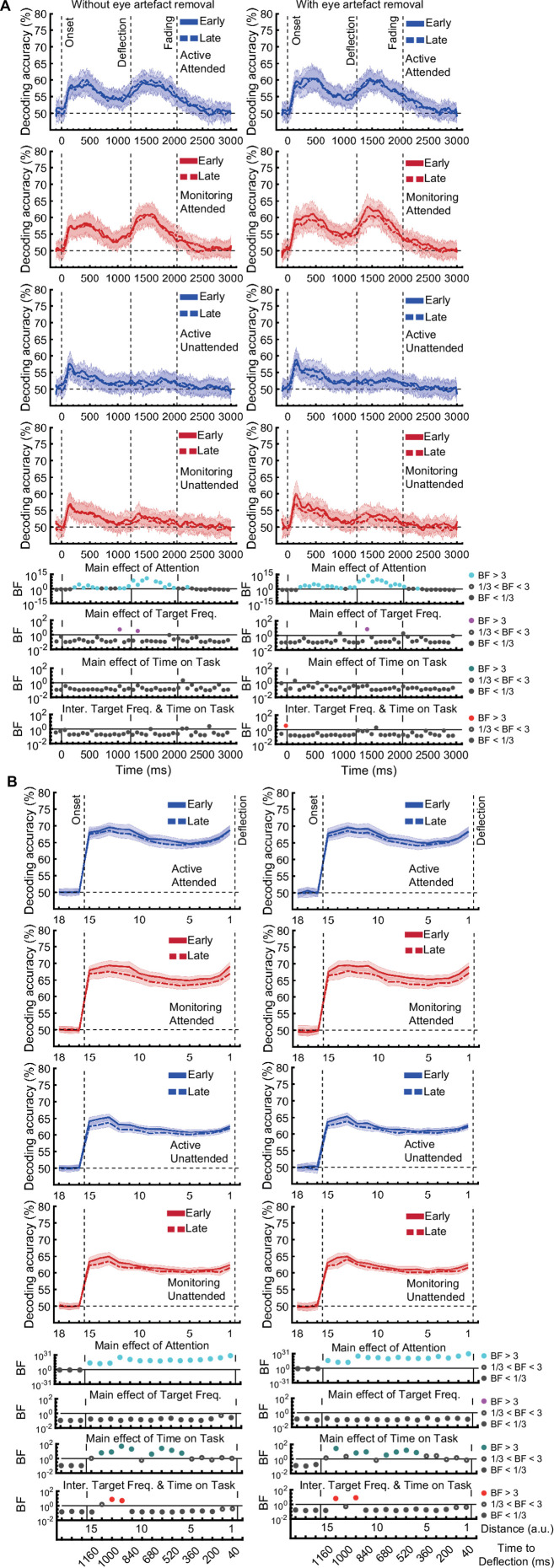


[**Results: Is informational brain connectivity modulated by Attention, Target Frequency and Time on Task?**]

[*Corrected text:* #1]

Results showed strong evidence (Bayes factor ANOVA, BF = 6.5e^3^) for higher informational connectivity for trials with Attended compared to Unattended dots, and moderate evidence for no effect of Target Frequency (Bayes factor ANOVA, BF = 0.11; *Figure 4B*). There was insufficient evidence to determine whether there was a main effect of Time on Task (Bayes factor ANOVA, BF = 0.72). There was evidence in the direction of the null for the two-way interactions between the factors (Bayes factor ANOVA, two-way Time on Task-Target Frequency: BF = 0.36; Time on Task-Attention: BF = 0.39; Target Frequency-Attention: BF = 0.15) and insufficient evidence regarding their three-way interaction (BF = 0.95). These results suggest that [--deleted text--] trials in which the dots are in the distractor (Unattended) colour, in which the attentional load is low, result in less informational connectivity between occipital and frontal brain areas compared to [--deleted text--] Attended trials. This is consistent with a previous study (Alnaes et al., 2015), which suggested that large-scale functional brain connectivity depends on the attentional load, and might underpin or accompany the decrease in information decoding across the brain in the unattended condition.

[*Original text:* #1]

Results showed strong evidence (Bayes factor ANOVA, BF = 6.3e^21^) for higher informational connectivity for trials with Attended compared to Unattended dots, and moderate evidence for higher connectivity in Active compared to Monitoring conditions (Bayes factor ANOVA, BF = 3.4; Figure 4B). There was insufficient evidence to determine whether there was a main effect of Time on Task (Bayes factor ANOVA, BF = 0.83). There was moderate evidence for no two-way and three- way interactions between the three factors (Bayes factor ANOVA, two-way Time on Task-Target Frequency: BF = 0.17; Time on Task-Attention: BF = 0.16; Target Frequency-Attention: BF = 0.15; their three-way interaction BF = 0.12). These results suggest that Monitoring conditions and trials in which the dots are in the distractor (Unattended) colour, in which the attentional load is low, result in less informational connectivity between occipital and frontal brain areas compared to Active conditions and Attended trials, respectively. This is consistent with a previous study (Alnæs et al., 2015), which suggested that large-scale functional brain connectivity depends on the attentional load, and might underpin or accompany the decrease in information decoding across the brain in these conditions.

[*Corrected figure: Figure 4B,C*]

**Figure 7:**
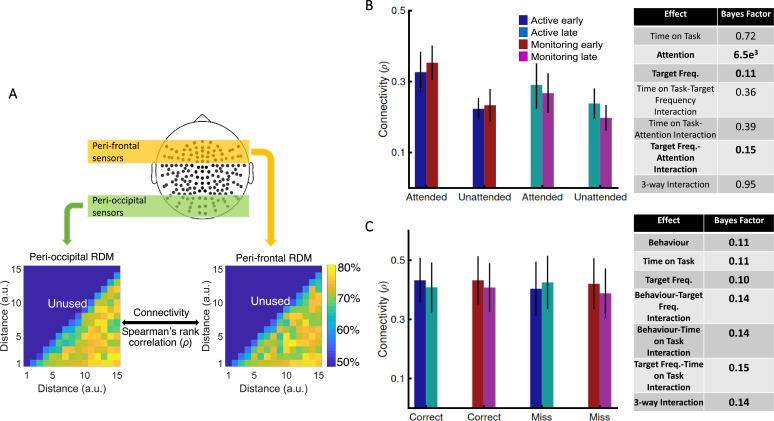


[*Original figure: Figure 4*]

**Figure 8:**
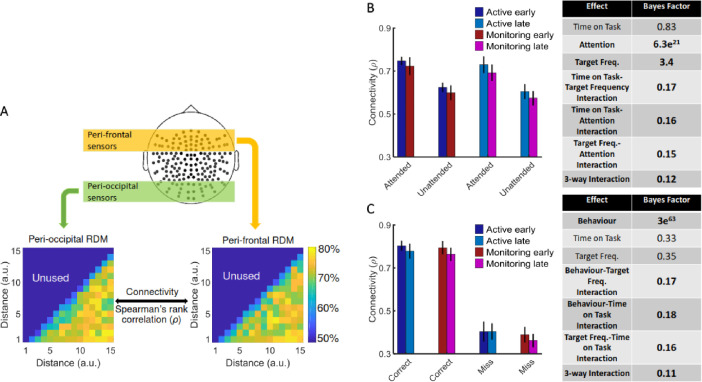


[*Corrected text:* #2]

There was moderate evidence for no difference in connectivity on *miss* compared to *correct* trials (Bayes factor ANOVA, BF = 0.11). In addition, there was moderate evidence for no effect of Time on Task and Target Frequency (BF = 0.11 and BF = 0.10, respectively), as well as for two-way and three-way interactions between the three factors (Bayes factor ANOVA, Behaviour-Target Frequency: BF = 0.14; Behaviour-Time on Task: BF = 0.14; Target Frequency-Time on Task: BF = 0.15; their 3-way interaction BF = 0.14). [--deleted text--] Therefore, in contrast to an auditory monitoring task which showed decline in univariate graph-theoretic connectivity before behavioural errors (Sadaghiani et al., 2015), we observed no change in informational connectivity on error. Note that, the number of trials is equalized across the 8 conditions in each of our analyses separately.

[*Original text:* #2]

There was strong evidence for less (almost half) connectivity on miss compared to correct trials (Bayes factor ANOVA, BF = 3e^63^). There was insufficient evidence to determine the effects of the Time on Task or Target Frequency (Bayes factor ANOVA, BF = 0.33 and BF = 0.35, respectively) and moderate evidence for a lack of two-way and three-way interactions between the three factors (Bayes factor ANOVA, Behaviour-Target Frequency: BF = 0.17; Behaviour-Time on Task: BF = 0.18; Target Frequency-Time on Task: BF = 0.16; their three-way interaction BF = 0.11). Weaker connectivity between occipital and frontal areas could have led to the behavioural misses observed in this study (Figure 1) as was previously reported in an auditory monitoring task using univariate graph- theoretic connectivity analyses (Sadaghiani et al., 2015), although, of course, these are correlational data and so we cannot make any strong causal inferences. These results cannot be explained by the number of trials as they are equalised across the eight conditions in each of the analyses separately.

[**Results: Is neural representation different on** ***miss*** **trials?**]

[*Corrected text:* #1]

On *correct* trials, the *distance* information for both Active and Monitoring conditions was above chance (Figure 5B left panels; BF >10^4^). For *miss* trials, the corresponding *distance* information was still above chance (Figure 5B right panels; BF >10^3^) but the direct comparison revealed that distance information dropped on miss trials compared to correct trials (*Figure 5B…*

[*Original text:* #1] On *correct* trials, the distance information for both Active and Monitoring conditions was well above chance (77%; BF >10). In contrast, for miss trials, the corresponding distance information was only just above chance (55%; BF >3 for all distances). The direct comparison revealed that distance information dropped considerably on miss trials compared to correct trials (*Figure 5B…*

[*Corrected text:* #2]

However, while the distribution of decoding accuracies for correct trials was centred around 60%, the decoding accuracies for individual miss trials were centred around 56%. We evaluated the difference in the distribution of classification accuracies between the two types of trials using Cohen’s d. Cohen’s d ranged from 0 to 2.5 across participants and conditions. 14 out of 21 subjects showed moderate (d>0.5) to large (d>0.8; Cohen, 1969) differences between the distribution of correct and miss trials in either Active or Monitoring condition or both. Therefore, although the miss trials vary somewhat in levels of information, only a minority of (<24%) miss trials are as informative as the least informative correct trials.

[*Original text:* #2]

However, while the distribution of decoding accuracies for correct trials was centred around 80%, the decoding accuracies for individual miss trials were centred around chance-level. We evaluated the difference in the distribution of classification accuracies between the two types of trials using Cohen’s d. Cohen’s d was approximately 3 or higher for all participants and conditions, indicating a large (d>2; Cohen, 1969) difference between the distribution of correct and miss trials. Therefore, although the miss trials vary somewhat in levels of information, very few (<7%) miss trials are as informative as the least informative correct trials.

[*Corrected figure: Figure 5B*]

**Figure 9:**
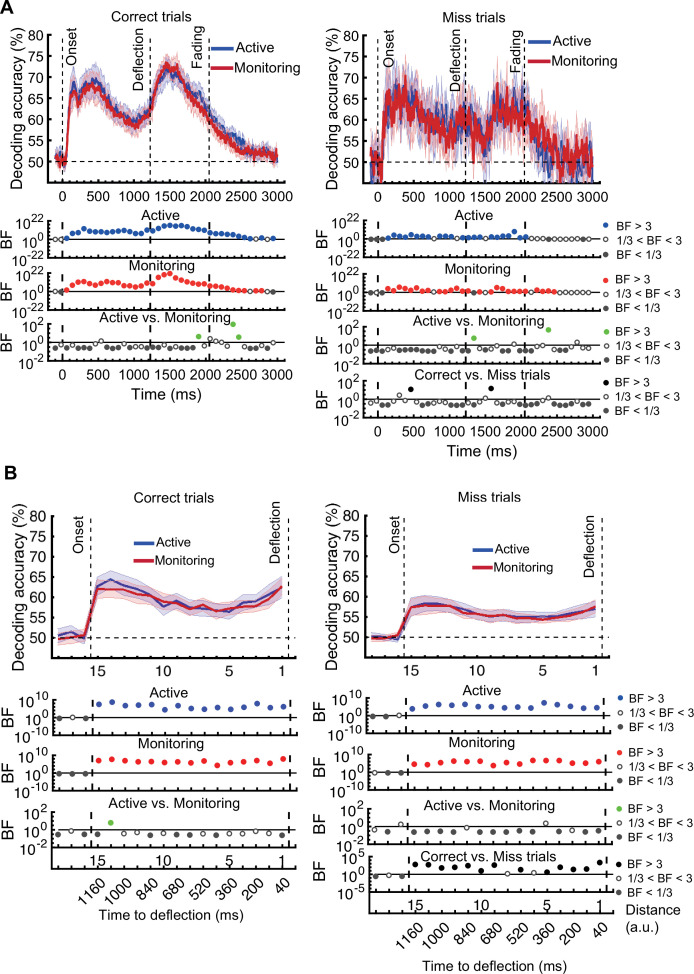


[*Original figure: Figure 5*]

**Figure 10:**
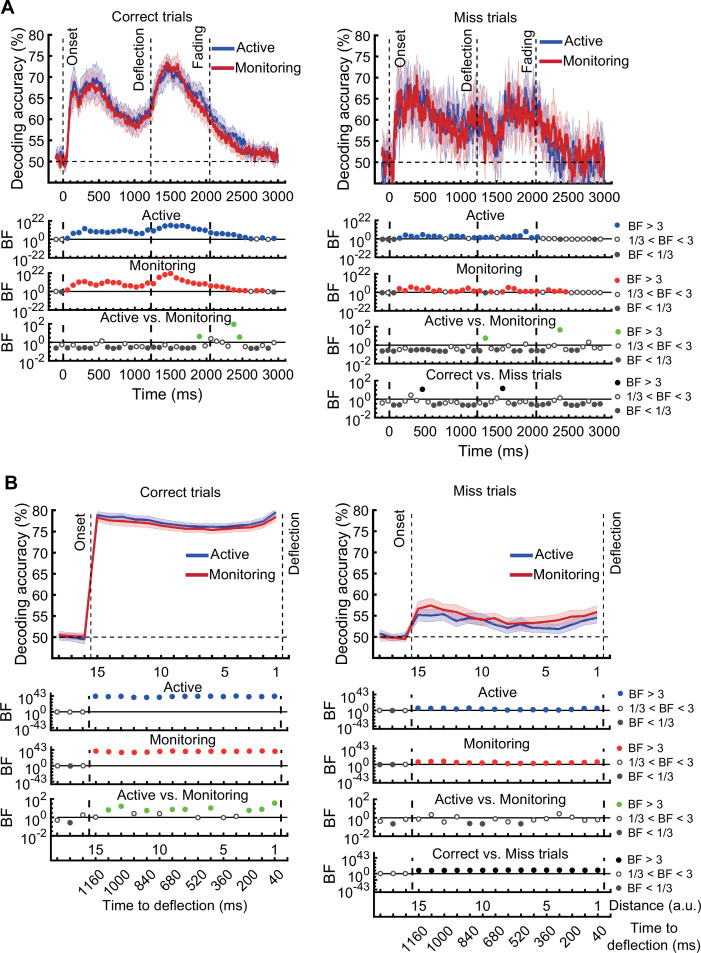


[*Corrected figure: Figure 5—figure supplement 1*]

**Figure 11:**
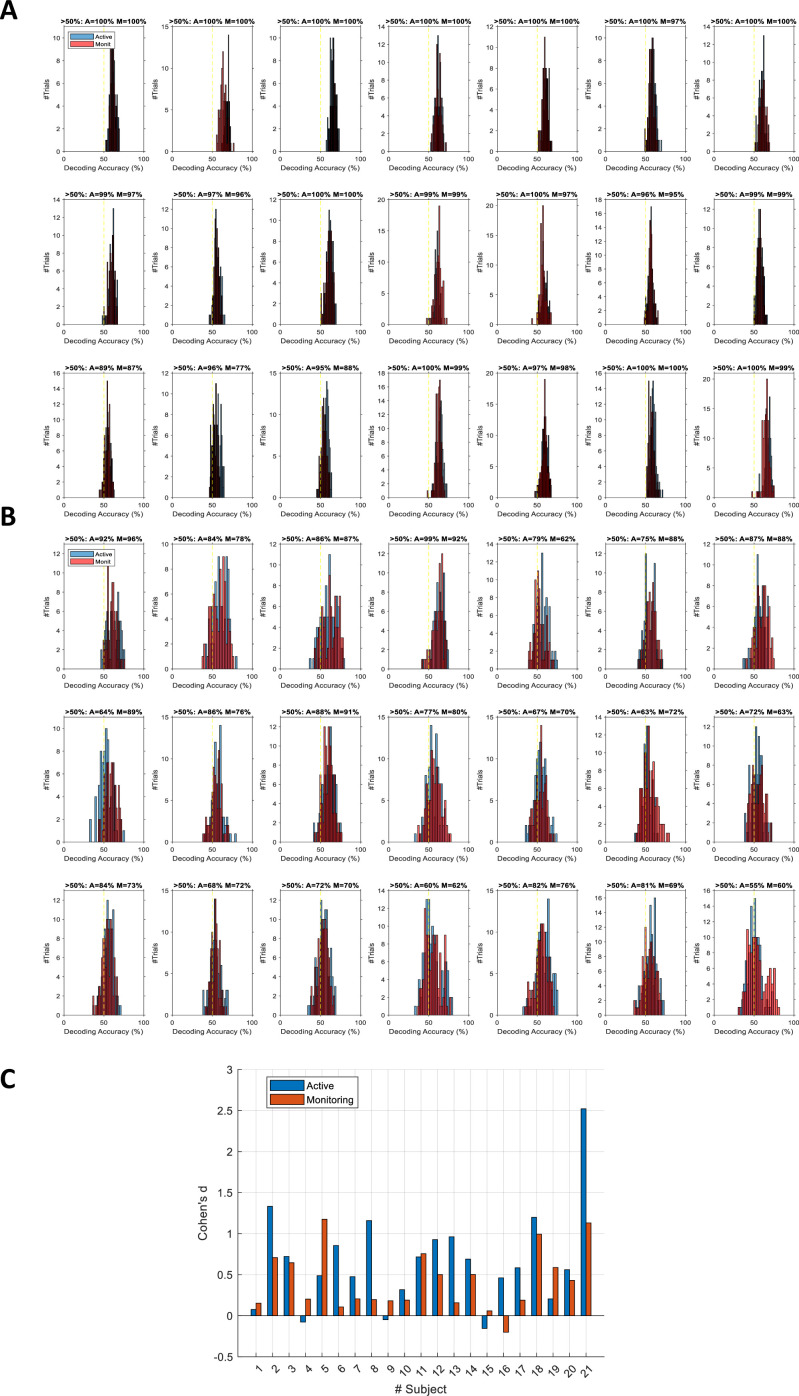


[*Original figure: Figure 5—figure supplement 1*]

**Figure 12:**
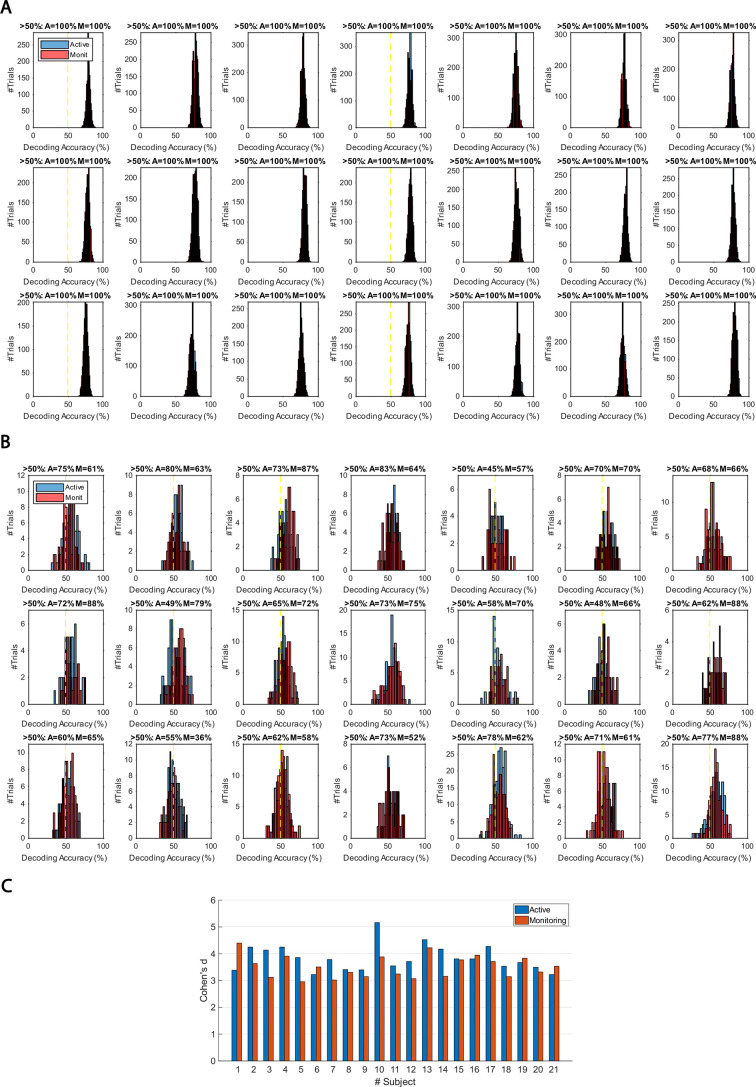


[*Corrected figure: Figure 5—figure supplement 2B]*

**Figure 13:**
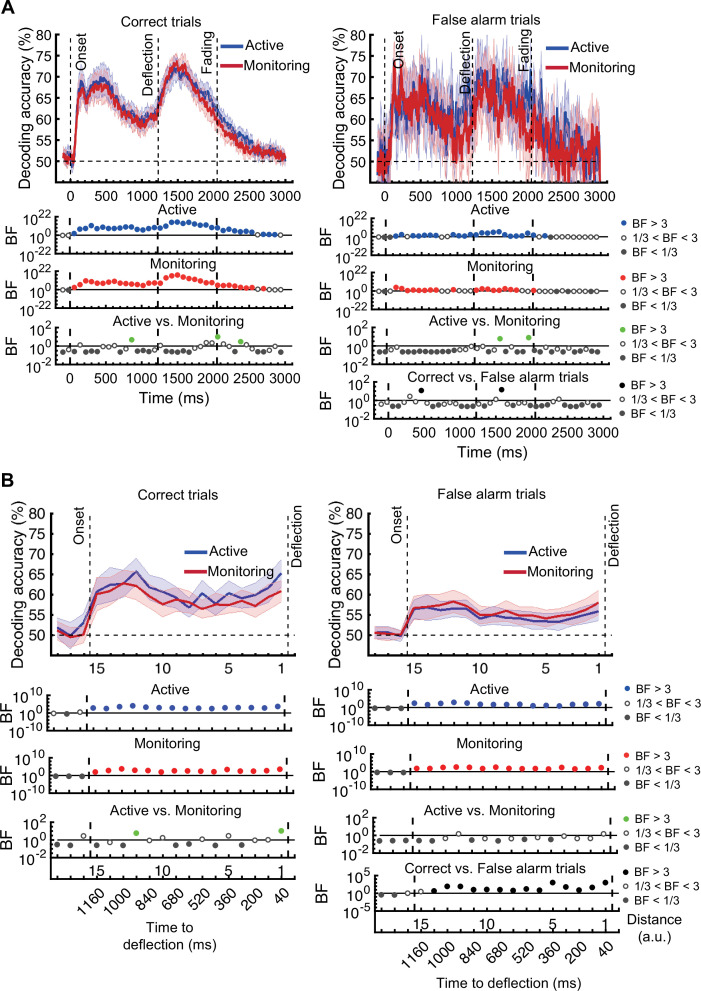


[*Original figure: Figure 5—figure supplement 2*]

**Figure 14:**
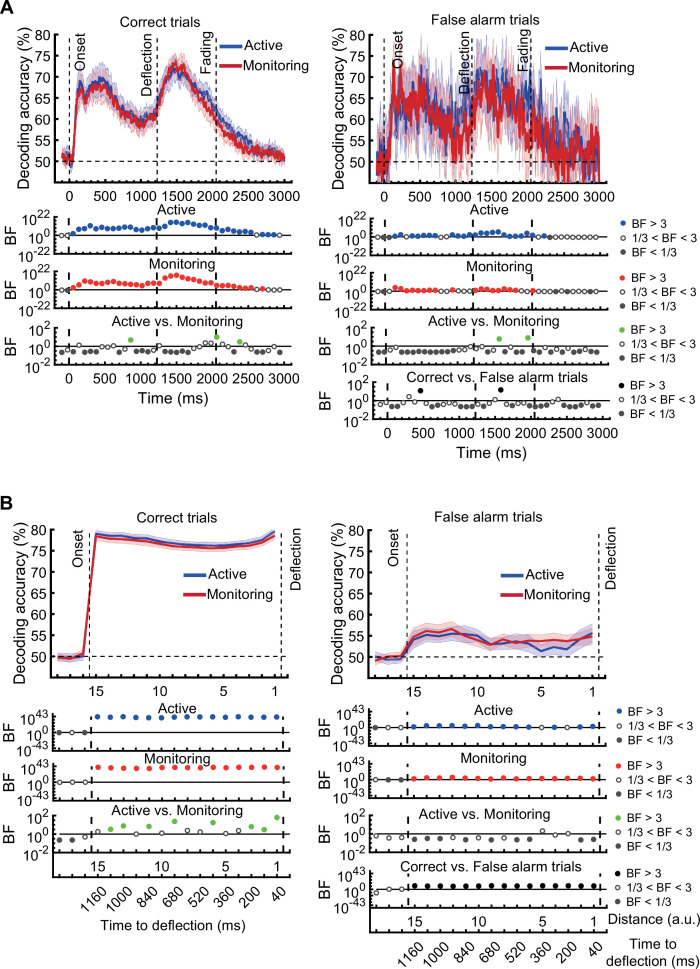


[**Results: Can we predict behavioural errors using neuroimaging?**]

[*Corrected text:* #1]

This was ~0.4 standard deviation below the average accuracy on the other participants’ validation (correct trial) sets (Figure 6C).

[*Original text:* #1]

This was ~1.5 standard deviation below the average accuracy on the other participants’ validation (correct trial) sets (Figure 6C).

[*Corrected text:* #2]

The prediction accuracy of behavioural outcome was above chance level (59% vs 50%; BF >10)….

[*Original text:* #2]

The prediction accuracy of behavioural outcome was above chance level (68% vs 50%; BF >10)…

[*Corrected text:* #3]

The accuracy increased to 65.4% as the dot approached the centre of the screen, with ~64% accuracy with still 800ms to go before required response.

[*Original text:* #3]

The accuracy increased to 85% as the dot approached the centre of the screen, with ~80% accuracy with still 800ms to go before required response.

[*Corrected text:* #4]

The prediction of behavioural outcome (Figure 6) was performed using the data from the whole data set. To test if prediction accuracy depended on the stage of the experiment, we performed the behavioural prediction procedure on data sets obtained from the first 5 (early) and the last 5 (late) stages of the experiment separately (Figure 6—figure supplement 1). There was no evidence for a change in the prediction power in the late vs. early blocks of trials.

[*Original text:* #4]

The prediction of behavioural outcome (Figure 6) was performed using the data from the whole data set. However, it is possible that the prediction would not be as accurate in later stages of the experiment (compared to the earlier stages) as the decoding performance of the distance information declined in general in later stages (Figure 3B). To test this, we performed the behavioural prediction procedure on data sets obtained from the first 5 (early) and the last 5 (late) stages of the experiment (Figure 6—figure supplement 1). There was strong evidence for a decline in the prediction power in the late vs. early blocks of trials. However, even with the decline in prediction accuracy, it is still possible to predict the behavioural outcome in the late blocks with well above-chance accuracy (up to 75%).

[*Corrected figure: Figure 6B,C,D*]

**Figure 15:**
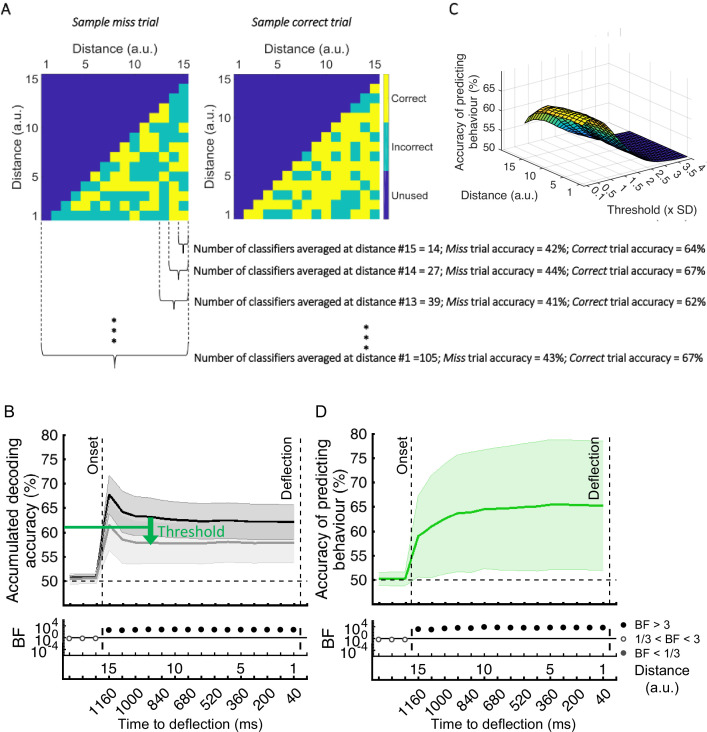


[*Corrected Figure 6 caption sections*]

Results showed highest prediction accuracies on the participant set at around the threshold of 0.4SD under the decoding level for correct validation trials, increasing at closer distances.

Results showed successful (~=59%) prediction of behavioural outcome of the trial as early as 80 ms after stimulus appearance.

[Original Figure 6 caption sections]

Results show highest prediction accuracies on the participant set at around the threshold of 1.5SD under the decoding level for correct validation trials, increasing at closer distances.

Results showed successful (~=70%) prediction of behavioural outcome of the trial as early as 80 ms after stimulus appearance.

[*Original figure: Figure 6*]

**Figure 16:**
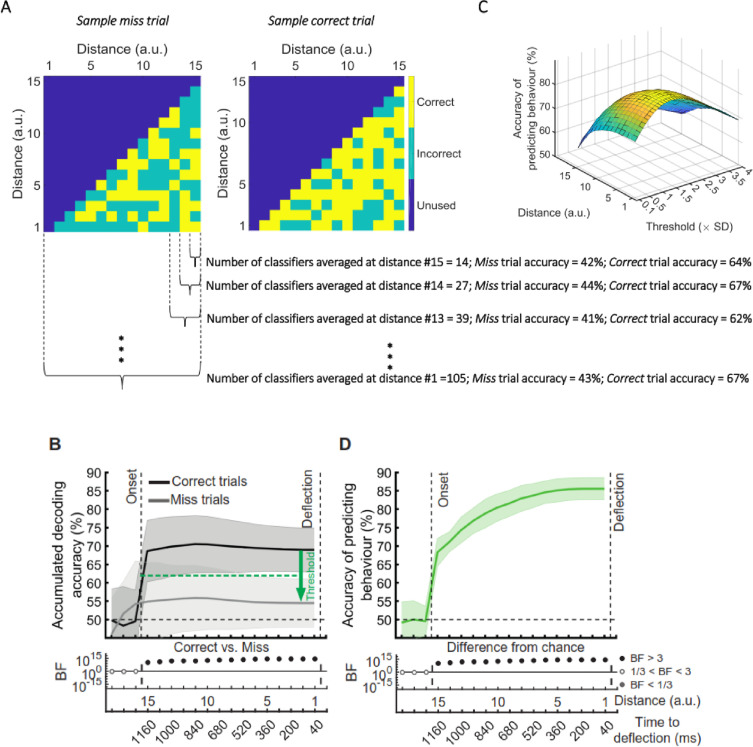


[*Corrected figure: Figure 6—figure supplement 1*]

**Figure 17:**
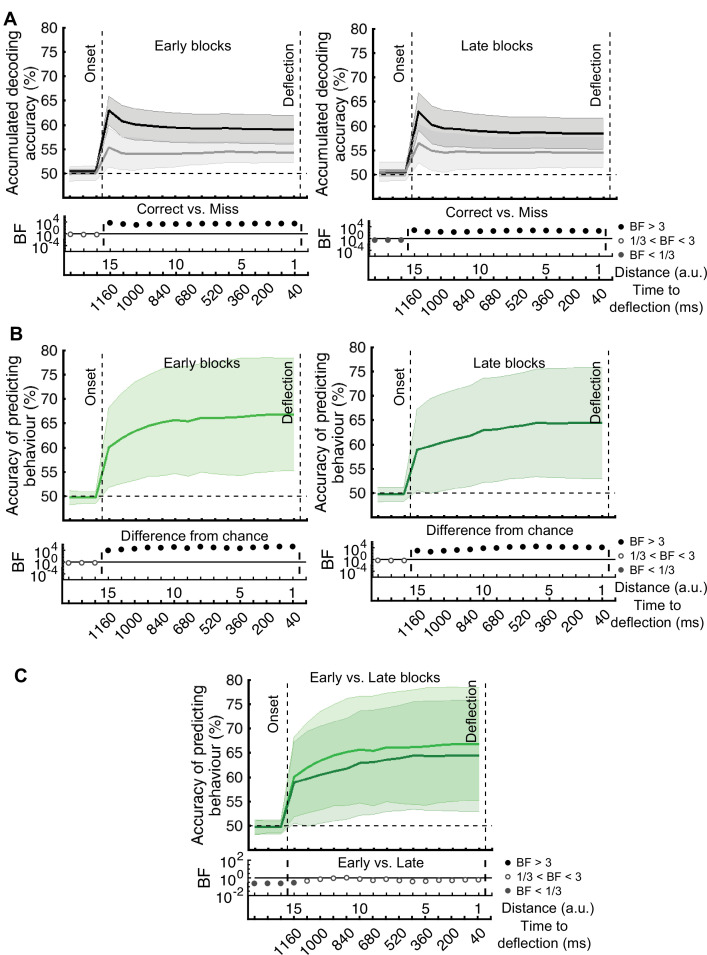


[*Original figure: Figure 6—figure supplement 1*]

**Figure 18:**
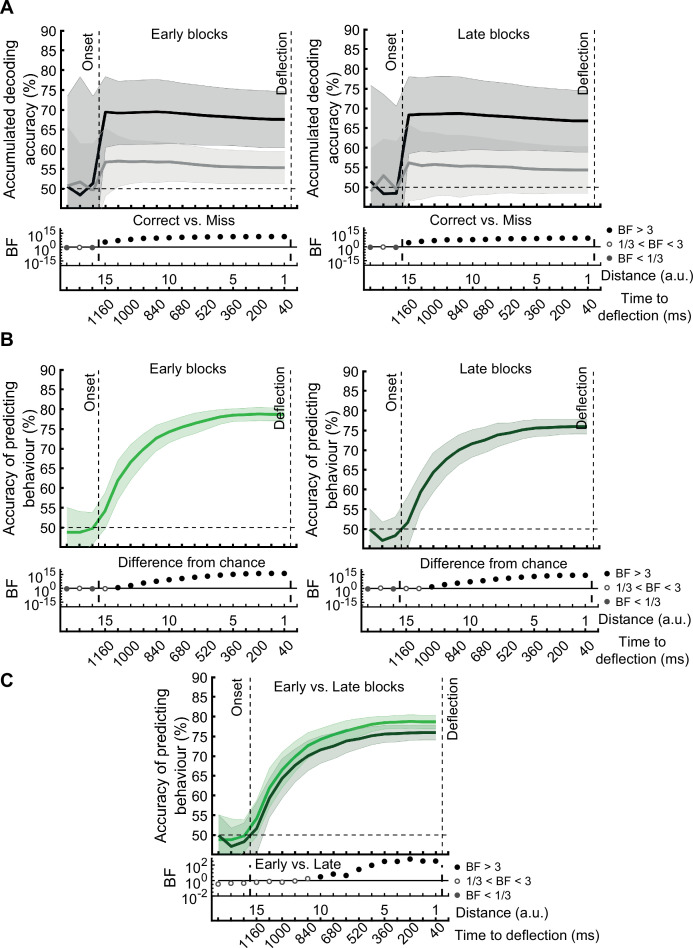


[**Discussion**]

[*Corrected text:* #1]

Using the sensitive analysis method of MVPA, we showed that neural coding was stronger for attended compared to distractor information. There was also one time-window where the interaction between the time on the task and target frequency affected decoding, with a larger decline in coding under monitoring conditions, which may reflect a neural correlate of the behavioural vigilance decrements.

[*Original text:* #1]

Using the sensitive analysis method of MVPA, we showed that neural coding decreased for relevant task information more when targets were infrequent than frequent (at longer task durations), providing neural correlates of the behavioural vigilance decrements.

[*Corrected text:* #2]

We also developed a novel informational brain connectivity analysis, which showed that the correlation between information coding across peri-occipital and peri-frontal areas varied with different levels of attention but did not change with errors.

[*Original text:* #2]

We also developed a novel informational brain connectivity analysis, which showed that the correlation between information coding across peri-occipital and peri-frontal areas varied with different levels of attention, target frequency and the time on the task.

[*Corrected text:* #3]

Finally, we utilised our recent error data analysis to predict forthcoming behavioural misses based on the neural data.

[*Original text:* #3]

Finally, we utilised our recent error data analysis to predict forthcoming behavioural misses with high accuracy based on the neural data.

[*Corrected text:* #4]

When targets were infrequent, modelling real-life monitoring situations, there was a strong behavioural drop in performance (i.e., vigilance effects in both accuracy and RT; *Figure 2*) and a hint in the brain activity data of a change in neural coding (namely one time-window showing evidence of an interaction between Target Frequency and Time on Task). We need more data to fully test this effect, however, our main finding is that of being able to use the difference in decoding between correct and miss trials to predict behaviour.

[*Original text:* #4]

When targets were infrequent, modelling real-life monitoring situations, the neural coding of crucial information about the task dropped, correlating with the decrease in behavioural performance (i.e., vigilance effects in both accuracy and RT; *Figure 2*).

[*Corrected text:* #5]

When people miss targets, they might process or encode the relevant sensory information *less* effectively than when they correctly respond to targets. [--deleted text--]

[*Original text:* #5]

One explanation for the decrease in decoding accuracy for task-relevant information could be that when people monitor for rare targets, they process or encode the relevant sensory information *less* effectively as the time passes, relative to conditions in which they are actively engaged in completing the task.

[*Corrected text:* #6]

[--deleted text--]

[*Original text:* #6]

For example, the drop in decoding over time for both Active and Monitoring that is seen in *Figure 3* might reflect some of the general changes in the characteristics of the recording hardware over the course of the experiment (e.g., the MEG system warming up), but our design allows us to dissociate these from the key vigilance effects we are interested in.

[*Corrected text:* #7]

In contrast, the task-relevant information of distance to object was affected by attention and was attenuated on errors.

[*Original text:* #7]

In contrast, the task-relevant information of distance to object was affected by attention, target frequency and time on task and was dramatically attenuated on errors.

[*Corrected text:* #8]

Our information-based brain connectivity method showed moderate evidence for no change in connectivity between correct and error trials. [--deleted text--] Informational connectivity is unaffected by differences in absolute levels of information encoding (e.g., lower coding on *miss* vs. *correct* trials). It could be sensitive to different levels of noise between conditions, but there was no evidence for that in this case. Apart from sensory information coding and sensory-based informational connectivity, which were evaluated here, there may be other correlates we have not addressed.

[*Original text:* #8]

Third, our information-based brain connectivity method showed weaker connectivity between the peri-frontal attentional network and the peri-occipital visual areas of the brain in the unattended and monitoring conditions (*Figure 4*), where participants encountered fewer targets relative to the other conditions. We also observed less connectivity between the same areas on *miss* vs. *correct* trials. Our connectivity is unaffected by the absolute lower level of information encoding in the brain on *miss* vs. *correct* trials, but it is possible that it is driven by noisier patterns of information encoding in *miss* (vs. *correct*) trials. Thus, the lower connectivity for *miss* vs. *correct* trials could result from (at least) two possible causes: the pair of regions representing two distinct sets of information (i.e., becoming in some sense less connected on misses) or the pair represent similar information that is distorted by higher levels of noise on misses. Apart from sensory information coding and sensory-based informational connectivity, which were evaluated here and provide plausible neural correlates for the vigilance decrement, there may be other correlates we have not addressed.

[*Corrected text:* #9]

This makes our analysis sensitive to different aspects of connectivity compared to conventional univariate analyses.

[*Original text:* #9]

This makes our analysis more sensitive for capturing subtle connectivity than the conventional univariate analyses.

[*Corrected text:* #10]

We statistically compared the two types of trials and showed a reliable advantage in the level of information contained at individual-trial-level in correct vs. miss trials.

Our error prediction results showed a reliable decline in the crucial task-relevant (i.e., distance to object) …

[*Original text:* #10]

We statistically compared the two types of trials and showed a large advantage in the level of information contained at individual-trial-level in correct vs. miss trials.

Our error prediction results showed a large decline in the crucial task-relevant (i.e., distance to object) …

[*Corrected text:* #11]

Here, we obtained up to 65% prediction (with a chance level of 50%), suggesting our method accesses relevant neural signatures of attention lapses, and may be sensitive in discriminating these.

[*Original text:* #11]

Here, we obtained up to 85% prediction (with a chance level of 50%), suggesting our method accesses more relevant neural signatures of vigilance decrements, or is more sensitive in discriminating these.

[*Corrected text:* #12]

We observed that the neural representation of critically relevant information in the brain [--deleted text--] was particularly poor on trials where participants missed the target.

[*Original text:* #12]

We observed that the neural representation of critically relevant information in the brain decreases over time, especially when targets are infrequent. This neural representation was particularly poor on trials where participants missed the target.

[*Corrected text:* #13]

We used this observation to predict behavioural outcome of individual trials and showed that we could predict behavioural outcome…

[*Original text:* #13]

We used this observation to predict behavioural outcome of individual trials and showed that we could accurately predict behavioural outcome…

[*Corrected text:* #14]

These results provide new insights about how momentary lapses in attention impact information coding in the brain and propose an avenue for predicting behavioural errors using novel neuroimaging analysis techniques.

[*Original text:* #14]

These results provide new insights about how vigilance decrements impact information coding in the brain and propose an avenue for predicting behavioural errors using novel neuroimaging analysis techniques.

[**Materials and Methods**]

[*Added text:* #1]

Each distance covered a time window of ~80ms (varied slightly as dot trajectories varied in angle) which consisted of 4 or 5 signal samples depending on which of the 15 predetermined distances was temporally closest to each signal sample and therefore could incorporate it.

[*Added text:* #2]

As within-trial autocorrelation in signals could inflate classification accuracy (signal samples closer in time are more similar than those farther apart), we ensured that in every cross-validation run and each distance, the training and testing sets used samples from distinct sets of trials. To achieve this, trials were first allocated randomly into 10 folds, without separating their constituent signal samples. This way, the 4 or 5 signal samples from within each distance of a given trial remained together across all cross-validation runs and were never split across training and testing sets.

[*Corrected text:* #1]

The optimal threshold was 0.4 (±0.07) times the SD below the decoding accuracy on the validation set across participants.

[*Original text:* #1]

The optimal threshold was 1.54 (±0.2) times the SD below the decoding accuracy on the validation set across participants.

[**Supplementary Materials**]

[*Corrected text: Source text file for Figure 3—figure supplement 1B*]

There was moderate or strong evidence that decoding of *distance* information for all attended conditions was greater than chance (50%, BF >3) across all 15 distance levels with the exception of distance 8 in the late monitoring condition (Figure 3—figure supplement 1B, left panels). There were also timepoints with greater than chance decoding for the unattended conditions but these were far less consistent (Figure 3—figure supplement 1B, right panels).

[*Original text: Source text file for Figure 3—figure supplement 1B*]

There was moderate or strong evidence that decoding of *distance* information for all conditions was greater than chance (50%, BF >3) across all 15 distance levels (Figure 3—figure supplement 1B).

